# Life Course Trajectories of Later-Life Cognitive Functions: Does Social Engagement in Old Age Matter?

**DOI:** 10.3390/ijerph14040393

**Published:** 2017-04-07

**Authors:** Sojung Park, Eunsun Kwon, Hyunjoo Lee

**Affiliations:** 1 George Warren Brown School of Social Work, Washington University, 1 Brookings Drive, Saint Louis, MO 63130, USA; 2 Center for Social Science, Seoul National University, 1 Gwanak-ro, Gwanak-gu, Seoul 08826, Korea; ek2218@columbia.edu; 3 Department of Social Work, Daegu University, 201 Deagudae-ro, Gyeongsan-si, Gyeongsangbuk-do 38453, Korea; hjlee7723@naver.com

**Keywords:** life-course perspective, cumulative disadvantage, cognitive function, social engagement, group trajectory

## Abstract

This study identified differential patterns of later-life cognitive function trajectories and examined to what extent life course factors and social engagement are associated with group trajectories. Data came from seven waves of the Health and Retirement Study (HRS 1998–2010; n = 7374; Observations = 41,051). Latent class growth analysis identified cognitive function trajectory groups, and multinomial logistic regression was used to examine the factors associated with group trajectories. Five heterogeneous trajectories were identified: stable high, stable moderate, stable low, high-to-moderate, and moderate-to-low. Findings suggest that, after adjusting for life course factors, individuals who became volunteers were more likely to belong to one of the two least vulnerable trajectories, stable high or high-to-moderate. Our findings suggest that, despite the cumulative life course factors evident in cognitive decline, social engagement in old age may serve as a potential protective resource.

## 1. Introduction

Identification of long-term risk and protective factors for cognitive function is an imperative task for scholars and policy makers on aging to address the economic and societal implications of increasing health care costs and needs [1]. Using life course approaches, research has suggested that age-related cognitive function changes may originate in early life experiences and there may be common risk factors [2]. 

We address three particular concerns. First, we examine multiple trajectories of cognitive function over time among older individuals. The life course perspective suggests that development of cognitive function is a dynamic, heterogeneous process; an aging individual’s health and behavior at any point not only reflects his/her accumulation of experiences but also shapes adaptations over the life course [3]. Growing longitudinal evidence has shown there are differential patterns of functional and mental health, but there is little knowledge on the multiple patterns of cognitive function in old age. 

Second, a large body of literature on later-life health disparities provides compelling evidence that adverse socioeconomic conditions during childhood and adolescence are a distal cause of cognitive decline and dementia [4,5,6]. However, most studies focus on a specific life stage or on one personal characteristic (e.g., race, gender, education). Little research has examined to what extent early-life adversity combined with later-life circumstances influence later-life cognitive function. 

Additionally, it has been suggested that the effects of negative life events (e.g., illness, death of loved one, involuntary relocation) that accumulate over the life course [7] are associated with cognitive decline in old age [8]. Conceivably, cognitive decline in old age reflects cumulative disadvantages from early life and adulthood, disruptive life events, and health changes in old age [9]. Existing studies, however, share limitations such as small samples or cross-sectional designs [10]; no study to date has comprehensively examined life course factors known to influence cognition in late life. 

Finally, much research has suggested that social engagement may serve a protective role in age-related cognitive decline [11]. However, empirical evidence is not conclusive due to weaknesses in current literature, including focusing on a single aspect of social environmental characteristics or lack of attention to changes in social engagement [11]. Importantly, many existing studies have focused on a single aspect of social engagement (e.g., social networks) or used an index of various social activities [12,13]. Beland and colleagues examined various aspects of social networks and social integration in cognitive decline over time and found that older individuals with higher levels of social networking with family (e.g., family ties, perceived support from family) and social integration (e.g., participation in community activities) maintained better cognitive function, although one aspect (having friends) was significantly related with less cognitive decline in women only [14]. Another study found social activity and social support were related to better cognitive function, but not social network size [15]. These findings demonstrate that associations between social relations and cognitive function may depend on the type of social engagement. Many prior studies, however, have been based on a short follow-up period [16,17,18] or did not examine the dynamic nature of social engagement [14,17,19,20]. It is important to examine how social engagement changes over time.

### Present Study

To our knowledge, no other study has examined comprehensively to what extent life course factors, life events, and health changes are associated with different patterns in cognitive function in old age over time or whether the widely recognized benefits of social engagement are still evident in old age. We have three research questions:

1. Can different group trajectories of cognitive function among older adults be identified during the 12-year observed period? We focus on life course factors in examining change trajectories of cognitive function in old age using a cognitive scale rather than a measure of cognitive impairment [21]. We expect to find disparate trajectory groups characterized as normal, age-vulnerable, and with pathological decline linked with neurological changes, and that cognition trajectories will further diverge as a result of heterogeneous developmental life-long processes.
2. Will the characteristics of later-life cognitive function trajectories vary based on a range of life course factors from childhood to old age? Based on the cumulative disadvantage perspective and prior research, we expect that disadvantages from childhood and adulthood as well as life events and health changes in old age will be associated with trajectory groups with lower levels of or declining cognitive function at baseline and over time.
3. To what extent is social engagement associated with trajectories of cognitive function? Based on previous literature on social engagement, we hypothesize that increased social engagement (frequency of engagement with social network and volunteering) over time will be associated with maintaining higher levels of cognitive function, while decreases in social engagement will be related to lower levels of cognitive function.

We hypothesize that, compared to social network engagement, a stronger pattern of association will emerge in volunteering since volunteering is expected to use more cognitive resources and contribute to delaying or preventing decline in cognitive function.

## 2. Materials and Methods

### 2.1. Data and Sample

Seven waves of data (1998–2010) from the Health and Retirement Study (HRS) in combination with data prepared by the RAND Center for the Study of Aging (RAND HRS Data, 2014) were used in this study. The HRS is a prospective cohort study conducted by the University of Michigan with support from the National Institute of Aging. The benefits of using the RAND HRS data include its collection of detailed health, behavioral, and demographic information and the use of bracketing methods to minimize nonresponse. Details of the multistage sample design, selection criteria, implementation, and response rates are available elsewhere [22]. In 1998, the original HRS sample was merged with the sample of Asset and Health Dynamics Among the Oldest Old (AHEAD) respondents and combined with two additional samples (Children of the Depression Era (CODA) and War Babies (WB)) making the data broadly representative of the entire household population of the United States aged 50 and older.

The initial analytical sample consisted of community-dwelling older individuals aged 65 or older (n = 11,036, Observations = 49,074). Respondents who did not provide information on childhood variables (8%) were excluded. Analyses showed these respondents’ cognitive function was similar to those in the sample, and analyses with imputed data (not shown) produced similar results. In order to best estimate trajectory of outcomes over time, the sample was limited to those who participated in at least three time points during the period of study. A series of hierarchical models were built to examine the role of theoretical constructs using the life course perspective. Since data with multiple imputation was not technically feasible for testing nested models in Stata, we dropped observations that had missing information on one of the covariates. We ran the same hierarchical models with multiple imputed data without testing nested modeling, and the results were essentially the same. Our final sample size was 7374 individuals with 41,051 observations. During the study period, 1721 (23.35%) respondents died. Including attrition status as a control variable during sensitivity tests introduced little change in effect sizes for the other variables, so attrition is not included in the models.

### 2.2. Measures

Cognitive function. In order to enhance generalizability of developmental trajectories, we examined the cognitive function of all respondents, including the most impaired. Following the approach adopted by previous research [23], we used a composite indicator from self-report and proxy since excluding proxies, as was done in most existing studies, undoubtedly would exclude highly impaired persons. 

For self-reports, determination of cognitive status was based on the Telephone Interview for Cognitive Status (TICS), a validated cognitive screening instrument patterned on the Mini-Mental State Examination [24]. TICS (0–35) includes immediate and delayed word recall, serial 7 backwards count, object identification, date naming, and president and vice president naming. Higher scores indicate higher cognitive functioning. Three levels of self-respondent cognitive function were constructed; scores of 0–12 = low cognitive function (0), scores ranging from 13 to 24 = moderate cognitive function (1), and scores of 25–35 = high cognitive function (2).

For proxies, a set of dichotomous variables indicated a problem in each of five areas, including respondent’s memory, ability to be left alone, propensity to hallucinate, tendency to get lost in familiar places, and tendency to wander off. Answers were summed ranging from 0 (no problems) to 5 (problems in all areas). For the final cognitive function indicator, we integrated the proxy cognitive function measures into the three levels of cognitive function derived from self-report: 3–5 problems = low cognitive function, 1–2 problems = moderate cognitive function, no problems (0) = high cognitive function. Detailed information on validity of the composite measure is provided in [23]. 

Life course factors. Guided by the life course perspective, we examined four factors:

1. ascribed factors, for which age was measured in years at baseline, gender (1 = female, 0 = male), and race/ethnicity (1 = white, 0 = non-white);
2. childhood socioeconomic status (SES) measured by parents’ education level (less than 8 years = 0, greater than 8 years = 1), family poverty status (1 = poor, 0 = not poor), and childhood health through retrospective reports in which respondents were asked to assess their health before the age of 16 (excellent = 1 to poor = 5);
3. achieved status in adulthood measured by years of education (0–17) and household income as a mean of log-transformed values across the observed period; and
4. life events and health conditions in adulthood measured by:
  - death of a spouse or divorce (0/1), nursing home admission (0/1), relocation (0/1)
  - chronic health conditions using the number of chronic diseases (high blood pressure, diabetes, cancer, lung disease, heart disease, stroke, psychiatric problems, and arthritis, 0 to 8) at baseline as well as change during the observed period (none experienced a decrease)
  - functional health based on the sum of activities of daily living (ADL) the respondent had problems with (difficulty with bathing, eating, dressing, walking across a room, and getting in or out of bed, 0–5) and instrumental activities of daily living (IADL) (using a telephone, taking medication, handling money, shopping, and preparing meals, 0–5). Three measures were used including number at baseline, increases during the observed period, decreases during that period.

Social engagement. Social engagement was measured using engagement with social network and volunteering. For social network, respondents were asked, “How often do you get together with any of your neighbors (or people in or near your facility) just for a chat or for a social visit?”. We standardized these reports by calculating a variable indicating days of social contact per week, with a range of 0–7; because the variable was highly skewed and had a non-linear distribution, we created a tertile indicator ranging from 0 to 2 with a higher value representing more engagement with three levels: baseline and increase or decrease. For volunteering, respondents were asked, “Have you spent any time in the past 12 months doing volunteer work for religious, educational, health-related or other charitable organizations?” (1 = yes, 0 = no) with three indicators: baseline and start or stop of volunteering during the observed period. 

### 2.3. Analysis

We used growth mixture modeling (GMM) to address the unobserved heterogeneity within data by extracting the number of latent classes and calculating the posterior probability of class membership for each individual [25]. This method assumes that the time-specific continuous measures of cognitive function may represent qualitatively different trajectories that exist in a population [26]. Specifically, latent class growth analysis (LCGA) was used, in which the variance and covariance of the intercept and the slope are fixed at zero and heterogeneity (i.e. the distribution of individual differences) is summarized by a finite set of unique polynomial functions, each corresponding to a discreet trajectory. Trajectory class membership is not known but inferred from the data based on posterior class membership probability [25,27,28].

First, models were run without covariates to establish the best-fitting model (and thus the number and shape of the trajectories) to identify distinct trajectories of cognition level. The analyses started with an intercept-only model, to which linear and quadratic growth factors were added to determine the forms of the growth model. A linear shape trajectory provided the better fit with the data than did a quadratic shape. The number of latent classes was determined by sequentially increasing the number of classes and examining fit statistics including Akaike information criteria (AIC), Bayesian information criteria (BIC) and entropy. We also conducted sequential tests to determine if the decrease in likelihood ratio with increase in class was statistically significant using the Lo-Mendell-Rubin test. Additionally, a proportion of each class (no less than 1%) was considered [29]. Models were tested with a number of start values to ensure the proper solution was obtained. Predicted classes of cognitive function were also checked with plots to assure classes represented within-individual trajectories rather than variations at each time point. 

Second, bivariate tests were conducted to examine characteristics of classes across life course factors. Third, multinomial logistic regression, with the most prevalent class as the referent, was used to determine the predictors of cognitive function trajectory class. We adopted a nested modeling strategy to evaluate the spectrum of multiple life course factors and experiences in social environment in old age. In the first model, we entered ascribed factors, early life conditions and adulthood education, then we added life events and health changes in old age. Finally, we added social network engagement and volunteering.

## 3. Results

### 3.1. Group Trajectory of Cognitive Function in Old Age

**Figure 1:**
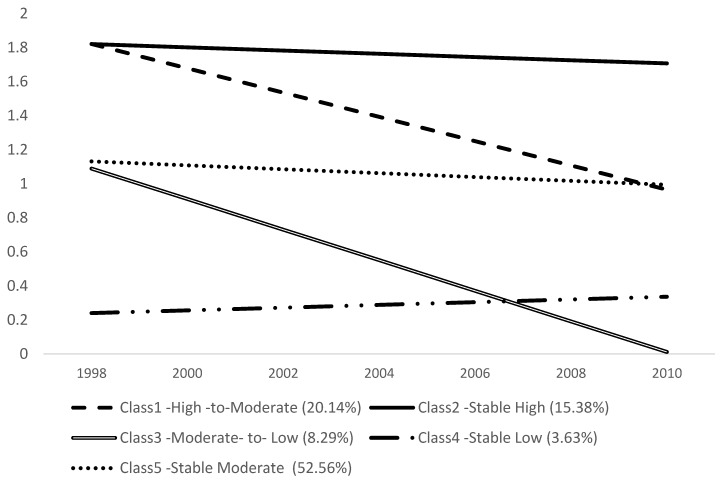
Cognitive function group trajectories in old age (65+ from the Health and Retirement Study (HRS) 1998–2010).

The change in the AIC, BIC, and Entropy coupled with a significant Lo-Mendel-Rubin likelihood ratio test suggested a five or six class model best fit the data (Table 1). We selected a five class solution because in six class the proportion of the one cluster was less than 1% of the sample. Figure 1 illustrates the trajectories of the five classes. Class 1 (20.14%) was termed as high-to-moderate (high initial cognitive function that declined to moderate); Class 2, stable high (15.38%) (consistently high level); Class 3 (8.29%), moderate-to-low (moderate initially that declined to low); Class 4, stable low group (3.63%) (consistently low); and Class 5, stable moderate, is the largest group (52.56%) (consistent moderate level).

### 3.2. Trajectory Groups of Cognitive Function by Life Course Factors

The trajectory groups significantly varied in all life course factors (Table 2). Clear patterns emerged among the three stable groups, high, moderate, low. Those in the stable high group were younger (Mean = 70.01), tended to be white (95.24%) and have had a relatively high level of self-rated health during childhood (1.81); they were also less likely to have parents with a low level of education (35.54%), and to have experienced family poverty (29.01%). Compared to other groups, those in the stable low group were more vulnerable: oldest (Mean = 78.79), non-white (38%), parents with less than eight years of education (88.4%), family poverty during childhood, lowest level of self-rated health in childhood (Mean = 2.18), fewest years of education (7.79 years) and lowest income (9.42). 

Among the two change groups, the moderate-to-low group appears to be the second most vulnerable group; they were the second oldest among all the groups (Mean = 77.12), more likely to have parents with little education (80.1% with <8 years), and had the second-worst level of self-rated health in childhood (Mean = 2.13); their education level was the second lowest (10.30 years) as was their income (9.76). 

The same hierarchical pattern of disadvantage holds in terms of life events and health. The stable low group was most likely to have experienced the death of a spouse or divorce (54.10%) and the highest incidence of nursing home admission (2.24%). At baseline, members of this group had the highest number of chronic conditions (Mean = –1.99) and functional limitations (Mean = 2.26). Relocation did not follow the pattern of other life course factors: the stable high group reported the highest level of relocation (39.86%), while the two disadvantaged groups, stable low and moderate-to-low groups, had relatively lower rates (22.01% and 27.17% respectively).

### 3.3. Role of Social Environmental Factors

Guided by the life course perspective, we built hierarchical models to examine to what extent various social engagement factors predict membership in different group trajectories of cognitive function (Table 3). The largest group, stable moderate, was used as the reference group. In model 1, ascribed factors, childhood factors and adulthood SES were entered. Partially consistent with the findings from the bivariate analyses, the results of multinomial logistic regression revealed ascribed factors and adulthood SES were strong predictors of trajectory group. None of the childhood factors except self-rated health was significantly associated with membership in any group. 

In model 2, major life events and changes in health conditions were added. Except for nursing home admission, no significant correlations were found between life events and the trajectory group. Nursing home admission was negatively associated with the high-to-moderate group (Relative Risk Ratio = 0.43) and positively related to the moderate-to-low group (RRR = 1.95). In terms of health, increases in functional change were strong predictors of trajectory group except for high-to-moderate: individuals whose functional limitations increased were more likely to be in the stable low (RRR = 3.26) or moderate-to-low group (RRR = 3.14). 

In model 3, social engagement factors were added. Patterns of association between social engagement and cognitive function trajectory emerged differentially. Changes in social network engagement were significantly associated with three trajectory groups after controlling for the baseline of social engagement: those who became more engaged were more likely to be high-to-moderate cognitive function (RRR = 1.24). Those who became less engaged over time were less likely to be in the stable-high group (RRR = 0.78). 

Significant effects of changes in volunteering were found across all cognitive function trajectories. After controlling for baseline, individuals who volunteered were more likely to be in the stable high or high-to-moderate group (RRR = 1.70 and RRR = 1.56). Those who stopped volunteering were more likely to be in the moderate-to-low group (RRR = 4.00).

## 4. Discussion

This study integrates the life course perspective and social engagement literature to empirically examine cognitive function in old age; to our knowledge, this study is the first to do so. Our findings are an important contribution to literature on cognitive decline and serve as much needed background for policy efforts to address increasing health care costs and the need for caregiving.

For the first research question, we asked whether different patterns of later-life cognitive function would emerge. Five heterogeneous trajectories were identified, with two different patterns of longitudinal changes: three groups had stable high, moderate, or low cognitive function during the observed period, while two groups had decreasing levels of function over time. 

From a pathological or abnormal aging perspective, the stable low group (3.65%) and the moderate-to-low group (8.29%) are particularly interesting. A large body of literature establishes a biological or neurological basis for poor cognitive function [30,31] and dementia [5]. The individuals in the stable low group may represent pathological aging considering they are the oldest subgroup and their cognitive function remains persistently low (0.2 to 0.3) over time. On the other hand, the moderate-to-low group had the second-oldest individuals and showed a steep decline of function over time, close to 0 by the end, which suggests their cognitive function declined to dementia or another type of cognitive pathology. 

For our second research question, we examined characteristics of the cognitive function trajectories by a range of life course factors. Guided by the cumulative disadvantage perspective, we generally expected to find that disadvantages over the life course are associated with groups whose cognitive function remains low or declines over time. 

Individuals in the stable-high groups tend to be white, have the fewest disadvantages in early life, and report the least stressful life events. On the other hand, older individuals in the stable low groups were likely to be most vulnerable: they tend to be non-white; they are also more likely to have disadvantageous childhood experiences, the lowest level of education in adulthood, the most stressful life events (e.g., death of a spouse, divorce, and reported high incidence of nursing home admission), and an increase in functional limitations in old age. The moderate-to-low group was among the most vulnerable groups, with advantages derived across the different life stages. Our findings show that later-life cognitive function trajectories align with the well–established pattern of socioeconomic disparity in old-age health. More importantly, they also show that the disparity in later-life cognitive function is associated with disadvantages across different life stages. 

Using the cumulative disadvantage perspective, we built a hierarchical life course model that enabled us to observe patterns of association with later-year cognitive function trajectories by life stages. Across all models, childhood factors were not associated with cognitive function in old age, while education in adulthood was strongly related with all cognitive function trajectories. The strong association between education and later-year cognitive function is consistent with previous studies [32]. The strong effect of education in adulthood seems to provide some support for the pathway model of the cumulative disadvantage perspective: childhood adversity itself does not necessarily determine long-term cognitive health; instead its effect may be either compounded by or ameliorated by education and income in later life stages. Although not the focus of the present study, empirically testing the extent to which education or social mobility in adulthood determines later-year cognitive trajectories in conjunction with the role of later-year social engagement is an important topic for future inquiry. 

Finally, we asked to what extent various aspects of later-year social engagement as potential protective factors are associated with cognitive function trajectories after adjusting for life course factors. Regression results indicate that changes in later-year social engagement over time are significantly associated with cognitive function trajectories. Our findings add stronger evidence of social engagement as a potential modifiable factor in the risk of cognitive decline, since we controlled for all life course factors and the baseline status of social engagement. As the first study to examine association between long-term change patterns of social engagement and group trajectories of cognitive function, our findings unfolded as we hypothesized. A diverging pattern of association emerged by changes in social engagement. Overall, increases in social engagement over time were associated with membership in the high-to-moderate and stable-high groups, whose cognitive health fell within a normal range of age-related decline and who were among the least disadvantaged. On the other hand, decreases in social engagement over time were associated with membership in the moderate-to-low group, the most vulnerable cognitive trajectory group. When older individuals experienced a reduced social network and became less engaged with neighbors in the community, they were more likely to be associated with the moderate-to-low group over time. The same pattern holds with changes in volunteering over time.

Our hypothesis regarding the stronger effect of volunteering as a risk/protective factor in cognitive decline was confirmed. Relative to the other two social engagement factors, the significant effect of changes in volunteering was found across all cognitive function trajectories. Importantly, individuals who volunteered were, over time, more likely to belong to the stable-high and high-to-moderate groups—the two least vulnerable trajectories. In contrast, when older individuals stopped volunteering over time, they were more likely to belong to the moderate-to-low group. This finding provides important implications for aging policy and programs, since it seems possible that even among individuals in the moderate-to-high group who experienced cumulative disadvantage over the life course, participation in volunteering may prevent or delay cognitive decline. The limited existing studies lend support to our findings. In a 6-month experimental study on volunteering among low-income older individuals, Carlson et al. [33] found evidence of volunteering as a modifiable risk factor against cognitive impairment. Neuroimaging test results showed that after older individuals participated in Experience Corps, a social program in which they volunteered in elementary schools to support children’s academic activities, they exhibited improvements in a range of brain activities. 

There are several limitations in our study. First, as expected we found a negative association between nursing home admission and the moderate-to-low group; Strong caution is required, however, in interpreting nursing home admission as a stressful life event causing cognitive function decline; it is entirely possible that cognitive decline led to nursing home admission. For a more accurate estimation, future studies should address the possibility of reverse causality.

We did not find any significant relation for relocation, although it appears the effects of relocation vary by group. Individuals in the stable high group are the healthiest and youngest and may be amenity-seekers when they relocate [34], while individuals in the two declining groups may have relocated to a more supportive living environment, in which case relocation would be more stressful. Our study was an initial attempt at incorporating life events as life course factors; as such our indicators may be not be sensitive enough to distinguish stressful events. Second, in this study it was not possible to examine physical activities. It would be ideal to examine the intersection of physical and social activity in relation to cognitive function over time. 

## 5. Conclusions

Despite these limitations, our findings illustrate that there are heterogeneous developmental trajectories in cognition in old age as a result of life course influences. Identification of vulnerable, at-risk groups for cognitive impairment contributes toward developing targeted policies and programs. Our findings suggest that despite the cumulative life course factors evident in cognitive decline, social engagement in old age may serve as a potential protective resource. 

## Figures and Tables

**Table 1:** Fit statistics for cognitive function group trajectories in old age (65+ from HRS 1998–2010).

Fit Statistic		Number of Classes				
	1	2	3	4	5	6
AIC ^a^		43,460.952	41,445.795	41,026.523	40,668.206	40,674.206
BIC ^a^		43,539.467	41,543.938	41,144.294	40,805.606	40,831.235
Entropy ^b^			0.81	0.74	0.75	0.75
LRT ^c^			<0.001	<0.001	<0.001	<0.05
Class proportion ^d^		Class 1 62.9% Class 2 37%	Class 1 12% Class 2 60% Class 3 27%	Class 1 54.2% Class 2 18.5% Class 3 15.3% Class 4 11.8%	Class 1 20.14% Class 2 15.38% Class 3 8.29% Class 4 3.63% Class 5 52.56%	Class 1 20.5% Class 2 2.9% Class 3 16.1% Class 4 9.1% Class 5 0.9% Class 6 50.2%

Note: HRS = Health and Retirement Study; BIC = Bayesian Information Criterion; LRT = Likelihood Ratio Test; AIC = Akaike Information Criteria. ^a^ A lower value suggests a better model fit. ^b^ A higher value suggests a better model fit. ^c^ Tests significance in the −2 times log-likelihood difference between the model with k and k−1 (H0) classes. ^d^ No less than 1% of total count in a class.

**Table 2:** Characteristics of cognitive function group trajectories (mean (standard deviation), or %) (n = 7374).

	Entire	Stable High	Stable Moderate	Stable Low	High to Moderate	Moderate to Low	Statistics
		15.38% (n = 1134)	52.56% (n = 3876)	3.63% (n = 268)	20.14% (n = 1485)	8.29% (n = 611)	
**Ascribed**							
Age (baseline)	73.44 (6.39)	70.01 (4.88)	74.17 (6.36)	78.79 (7.11)	71.67 (5.31)	77.12 (6.52)	F(47,369) = 249.84 ***^,a^
White	87.33	95.24	86.51	61.94	91.99	77.74	$x^{2}$(4) = 302.55 ***
Women	58.64	61.46	54.95	64.93	61.35	67.43	$x^{2}$(4) = 53.76 ***
**Child hood SES**							
Parent education (<8 years)	58.49	35.54	63.70	88.43	47.74	80.01	$x^{2}$(4) = 586.65 ***
Family poor	34.28	29.01	35.55	45.90	31.31	37.91	$x^{2}$(4) = 42.40 ***
Self-rated health	2.02 (1.01)	1.81 (1.01)	2.06 (1.01)	2.18 (0.98)	1.99 (1.00)	2.13 (1.05)	F(47,369) = 5.66 ***
**Adulthood SES**							
Education	11.92 (3.38)	13.46 (2.84)	11.62 (3.19)	7.79 (4.16)	12.96 (2.85)	10.30 (3.78)	F(47,369) = 270.11 ***
Income ($, log mean value)	10.15 (0.74)	10.55 (0.74)	10.06 (0.72)	9.42 (0.63)	10.36 (0.67)	9.76 (0.68)	F(47,369) = 263.69 ***
**Life events and health**							
Widowed/divorced	34.66	24.34	35.66	54.10	29.23	52.05	$x^{2}$(4) = 200.77 ***
Nursing home admission	1.33	1.41	1.34	2.24	0.47	2.95	$x^{2}$(4) = 22.26 ***
Relocation	33.84	39.86	32.79	22.01	36.84	27.17	$x^{2}$(4) = 55.05 ***
**Chronic condition (baseline)**	1.73 (1.25)	1.45 (1.12)	1.82 (1.27)	1.99 (1.38)	1.59 (1.20)	1.88 (1.25)	F(47,369) = 28.17 ***
No change	30.07	27.43	30.91	36.19	28.28	31.26	$x^{2}$(4) = 12.51 **
Increased	69.93	72.57	69.09	63.81	71.72	68.74	
**Functional limitations (baseline)**	0.50 (1.37)	0.20 (0.85)	0.50 (1.33)	2.26 (2.87)	0.25 (0.90)	0.81 (1.75)	F(47,369) = 153.09 ***
No change	48.71	71.52	46.10	15.76	56.16	19.31	$x^{2}$(8) = 654.07 ***
Increased	45.46	24.25	47.08	72.01	39.66	76.92	
Decreased	5.83	4.23	6.81	12.31	4.18	3.76	

Notes: A Significance level of *p*-value * < 0.05, ** *p*-value < 0.01, *** *p*-value < 0.001; SES = Socioeconomic Status.

**Table 3:** Life course factors with cognitive function trajectory groups in old age.

	Model 1 Ascribed+Childhood+Achieved				Model 2 Life Events+Health in Old Age				Model 3 Social Engagement in Old Age			
	Stable High ^a^	Stable Low	High to Moderate	Moderate to Low	Stable High	Stable Low	High to Moderate	Moderate to Low	Stable High	Stable Low	High to Moderate	Moderate to Low
	*Relative Risk Ratio*				*Relative Risk Ratio*				*Relative Risk Ratio*			
**Life course factors**												
**Childhood SES**												
**Parent education (<8 years)**	1.14	0.78	1.06	0.80	1.13	0.81	1.08	0.82	1.13	0.82	1.08 ***	0.83
Family poor	1.02	0.95	1.00	0.96	1.03	0.93	1.02	0.97	1.03	0.94	1.01	0.97
Self-rated health	0.88 ***	0.96	1.02	1.00	0.90 *	0.93	1.03	0.99	0.90 **	0.94	1.02	0.99
**Adulthood SES**												
Education	1.15 ***	0.82 ***	1.11 ***	0.92 ***	1.14 ***	0.83 ***	1.11 ***	0.92 ***	1.12 ***	0.84 ***	1.09 ***	0.92 ***
Income ($, log mean value)	1.83 ***	0.56 ***	1.44 ***	0.80 **	1.82 ***	0.61 ***	1.44 ***	0.85	1.75 ***	0.65 **	1.39 ***	0.86
**Life events and health**												
Widowed/divorced					1.15	0.85	1.12	1.16	1.14	0.88	1.10	1.15
Nursing home admission					1.51	0.94	0.43 *	1.95 *	1.51	1.07	0.43 *	2.18 **
Relocation					0.97	0.91	0.93	0.98	0.96	0.89	0.93	0.97
**Chronic condition (baseline)**					0.90 **	0.82 ***	0.93	0.91 *	0.91 **	0.81	0.94 *	0.91 *
Increased(ref. no change)					1.03	0.83	0.93 *	0.93	1.04	0.81 ***	0.99	0.90
**Functional limitations (baseline)**					0.99	1.34 ***	0.94	1.07 *	1.00	1.32 ***	0.96	1.07 *
Increased (reference. no change)					0.54 ***	3.26 ***	0.94	3.14 ***	0.59 ***	3.09 ***	0.98	2.79 ***
Decreased					0.70	1.34	0.80	0.82	0.71	1.35	0.80	0.76
**Social Engagement**												
Meet Frequency (baseline)									1.08	0.96	1.13 **	0.93
Increased (ref. no change)									1.11	0.92	1.24 *	0.76 *
Decreased									0.78 **	0.74	0.96	1.07
**Volunteered (baseline)**									1.70 ***	0.28 *	1.56 ***	0.32 ***
**Become volunteering** **(ref. no change)**									1.34 *	0.28 *	1.30 *	0.46 *
Become non-volunteering									0.66 ***	2.78	0.80 *	4.00 ***
**Constant**	0.28	0.36	0.14 **	0.06 **	0.18	1.01	0.14 **	0.11 *	0.24	0.73	0.16 *	0.14
	LR chi2(32) = 2215.61 ***				LR chi2(60) = 2242.73 ****				LR chi2(84) = 2005.65 ***			
**∆χ^2^**	-				371.18(32) ***				109.42(24) ***			

Note: All ascribed factors were controlled for in all models. Significance level of * *p*-value <0.05, ** *p*-value < 0.01, *** *p*-value < 0.001. ^a^ Reference = Moderate Stable Group.
